# Beyond bacterial vaginosis: vaginal lactobacilli and HIV risk

**DOI:** 10.1186/s40168-021-01183-x

**Published:** 2021-12-10

**Authors:** Eric Armstrong, Rupert Kaul

**Affiliations:** 1 Department of Medicine, University of Toronto, Toronto, Canada; 2 University Health Network, Toronto General Hospital, Immunodeficiency Clinic, Toronto, Canada

**Keywords:** Vaginal microbiota, HIV, immunology, bacterial vaginosis, *Lactobacillus*

## Abstract

HIV incidence continues to be unacceptably high in Eastern and Southern Africa, with women disproportionately affected. An increased per-contact risk of HIV acquisition among African, Caribbean, and other Black (ACB) women has been associated with the higher prevalence of bacterial vaginosis (BV) in these communities, wherein the vaginal microbiota is predominated by diverse pro-inflammatory anaerobic bacteria. However, while the vaginal microbiota in BV-free women is typically predominated by one of several different *Lactobacillus* spp., the degree of HIV protection afforded by a *Lactobacillus*-predominant vaginal microbiota also varies considerably. Specifically, *L. crispatus* is associated with an immunoregulatory genital immune environment, exclusion of BV-associated bacteria, and reduced HIV risk. In contrast, less HIV protection or exclusion of BV-associated bacteria and fewer immune benefits have been associated with *L. iners*—which is unfortunately the most common *Lactobacillus* species among ACB women. These species-specific clinical differences are underpinned by substantial genomic differences between *Lactobacillus* species: for instance, the much smaller genome of *L. iners* lacks the coding sequence for D-lactic acid dehydrogenase and cannot produce the D-lactate isomer that enhances HIV trapping in mucus but encodes for epithelial cell toxins and stress resistance proteins that may enhance bacterial survival in the context of microbiota and environmental fluctuations. While more studies are needed to elucidate whether differences in HIV protection between *Lactobacillus* species are due to direct genital immune effects or the exclusion of proinflammatory BV-associated bacteria, the current body of work suggests that for BV treatment to succeed as an HIV prevention strategy, it may be necessary to induce a vaginal microbiota that is predominated by specific (non*-iners*) *Lactobacillus* species.

Video abstract

**Supplementary Information:**

The online version contains supplementary material available at 10.1186/s40168-021-01183-x.

## Introduction

**Fig. 1:**
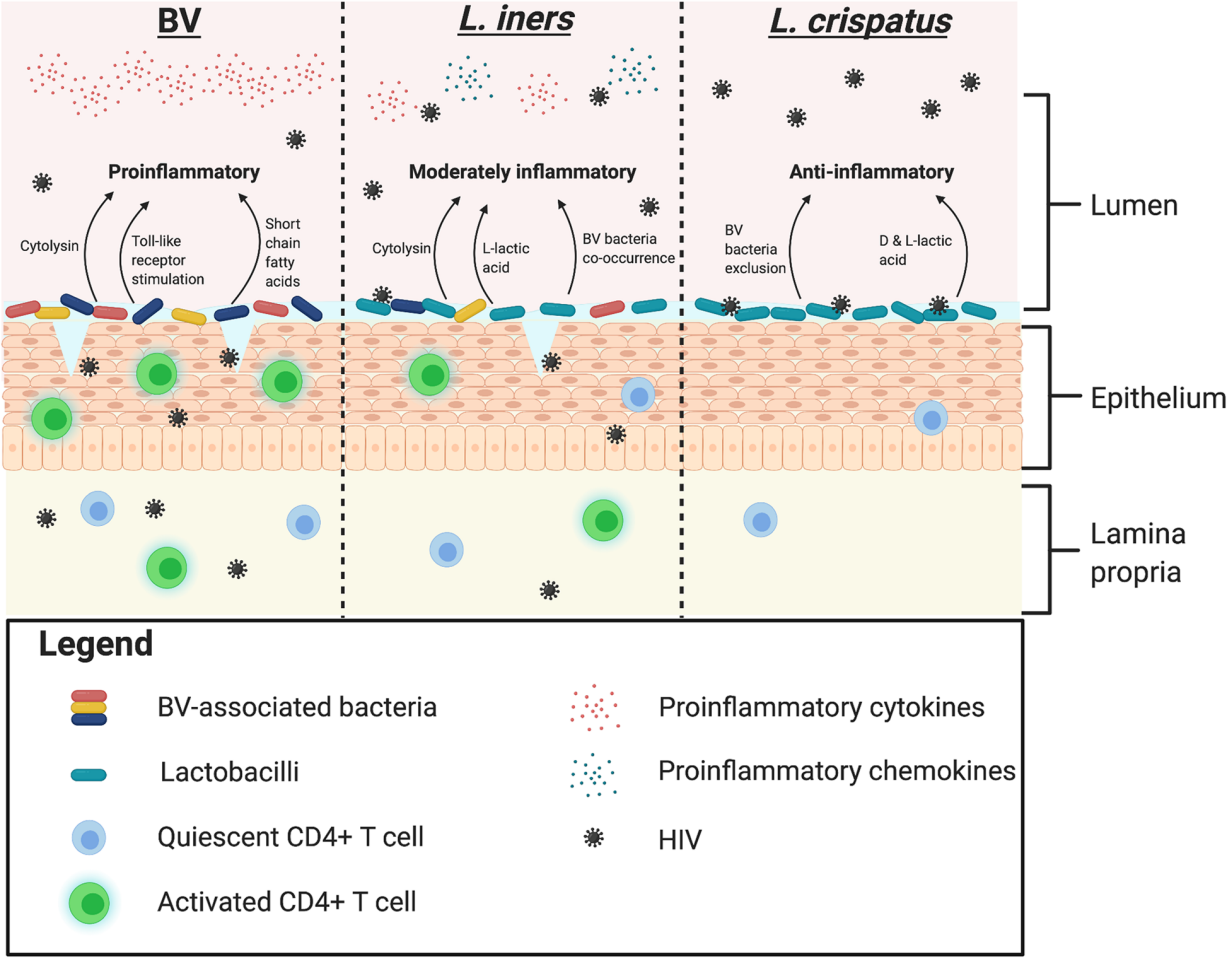
Potential mechanisms underpinning the differential levels of protection against HIV conferred by *L. iners* and *L. crispatus*. Created with Biorender.com

HIV remains a major health issue worldwide, particularly in the eastern and southern regions of sub-Saharan Africa (SSA) [1]. In contrast to the HIV epidemic in North America and Europe, where risk is disproportionately high in men who have sex with other men (MSM), in SSA, the majority of people infected are women [2]. Recent research has linked the elevated HIV risk that is seen in women from sub-Saharan Africa, and more broadly in all African, Caribbean, and other Black (ACB) women, to the composition of the vaginal microbiota. Specifically, there is an increased prevalence of bacterial vaginosis (BV) in ACB women [3, 4], which is characterized by high microbial diversity, and BV has been consistently linked to genital mucosal inflammation and elevated HIV risk [5]. However, as is the focus of this review, even in the absence of BV, the composition of the vaginal microbiota may play a key role in HIV susceptibility (Fig. 1).

In the absence of BV, the vaginal microbiota is typically predominated by one of several species of *Lactobacillus*, most commonly *L. crispatus* or *L. iners* [4]. A *Lactobacillus*-predominant microbiota is associated with reduced rates of several adverse reproductive health outcomes [6, 7]. However, although *Lactobacillus* predominance is broadly associated with protection against HIV, the degree of protection afforded varies substantially based on the dominance of different *Lactobacillus* species, likely due to heterogeneity in their genital immune impact and their ability to competitively exclude BV-associated bacteria. This review will focus on the mechanisms by which different species of *Lactobacillus* differentially impact HIV risk, illustrating the potential need for microbiota-focused HIV prevention strategies that not only treat BV, but also induce a vaginal microbiota that is predominated by non-*iners* species of *Lactobacillus*.

### The vaginal microbiota: a unique human niche

The human vaginal microbiota has evolved to serve the dual roles of protecting the female urogenital tract from infection while facilitating the implantation and development of a semi-allogenic fetus [8, 9]. In direct contrast to the microbiota of the human gut, where increased diversity of the microbiota provides a number of health benefits [10], in the vagina, it is a low diversity microbiota that confers protection against viral infections such as HIV and herpes simplex virus type 2 (HSV-2), as well as classical sexually transmitted infections (STIs) such as gonorrhea and chlamydia [6]. In a low-diversity state, the vaginal microbiota is typically predominated by one of the *Lactobacillus* species, most commonly either *L. crispatus*, *L. iners*, *L. gasseri*, or *L. jensenii* [4]. In the absence of *Lactobacillus* predominance, the vaginal microbiota is typically characterized by a diverse population of Gram-positive, Gram-negative, and Gram-variable bacteria [11], which has been linked to elevated risk of adverse reproductive health outcomes, including HIV/STI acquisition [12] and, less consistently, preterm birth [7, 13, 14].

Early studies relied completely on the presence of genital symptoms for a diagnosis of BV, defined by the Amsel criteria as the presence of at least 3 of: vaginal pH > 4.5; a thin, white/yellow discharge; the presence of clue cells on a bedside wet prep; and/or the release of a fishy odor after addition of 10% potassium hydroxide to vaginal secretions [15]. Recognition that BV could be asymptomatic or pauci-symptomatic, characterized by alterations in vaginal bacteria without all clinical findings, led to the development of the Nugent criteria that are used in many cohort-based studies [16]. Here, BV is defined by the presence of Gram-negative and Gram-variable bacteria and the absence of Gram-positive rods (lactobacilli) on a Gram stain. Symptom-independent BV characterization has been greatly enhanced by the introduction of 16S rRNA gene sequencing, which has allowed for detailed characterization of the bacterial genera and species associated with BV. McKinnon and colleagues termed the name “molecular BV” to describe a vaginal microbiota characterized by a paucity of *Lactobacillus* species and predominance by anaerobic gram-positive and gram-variable bacteria; however, there is not an established definition of molecular BV [4, 11].

Similar to other human microbial niches, the vaginal microbiota is impacted by a variety of endogenous and exogenous factors, and these may potentially confound microbiome associations of HIV risk: these include phase of the menstrual cycle [17], sexual activity (particularly the introduction of a new male sexual partner), the use of sex toys, the use of vaginal lubricant [18], smoking [19], contraceptive use (both hormonal and non-hormonal) [20, 21], and race [4]. The latter may be particularly important in the context of race-based differences in HIV susceptibility, given that a large cross-sectional study using 16S-based sequencing to investigate the vaginal microbiota of North American women found BV by 16S rRNA gene sequencing in ~40% of black women as opposed to just 10% of white women [4]. While it is unclear whether these race-based differences in the vaginal microbiota are driven by behavioral factors, they have been demonstrated in cohort studies that attempt to control for potential confounders such as douching and number of sexual partners [22–24]. Furthermore, because condomless sex is both a key determinant of HIV risk and also linked to disruptions in the vaginal microbiota [25], this may confound the relationship that has been described between the vaginal microbiota and HIV acquisition. While the vaginal microbiota remains independently associated with HIV risk in studies where sexual behaviors were explicitly controlled for [26] or did not differ between women with different vaginal bacterial community state types [27], these potential confounders (particularly the link between the vaginal microbiota and recent sex) highlight the importance of recapitulating these findings in other cohorts and ideally of demonstrating causation through microbiota-focused randomized clinical trials of HIV prevention.

### Clinical approaches to BV

BV management is hampered by both the lack of efficient screening tools for BV, and the inadequacy of current clinical treatment options. The standard of care is a 5- to 7-day course of oral or topical antibiotics, most commonly metronidazole [28, 29]. However, initial success is under 80% [30] and recurrence rates are ~40% within 3 months of treatment [31]. The inadequacy of current medical approaches means that alternative treatment modalities are commonly used, including vaginal probiotics and topical agents such as lactic acid, boric acid, hydrogen peroxide, and acetic acid; in addition, an intriguing recent study suggests that vaginal microbiota transplant may be an option in the future [31–33].

Given that BV is defined based on the vaginal microbiota composition, the application of vaginal probiotics—defined as “live microorganisms which, when administered in adequate amounts, confer a health benefit on the host” [34]—would appear to be an intuitive treatment approach. Early efforts to treat BV with *Lactobacillus*-based probiotics did not provide sufficient evidence that probiotic treatment was superior to standard antibiotic treatment, perhaps due to the use of *Lactobacillus* species that are not generally part of the natural vaginal flora [35]. Therefore, there is now a greater effort to develop and test probiotics that contain vaginal-resident *Lactobacillus* species and strains [36]. Most pertinent, a recent randomized, placebo-controlled trial demonstrated that the vaginal application after standard BV treatment of a topical *L. crispatus*-based live biotherapeutic (LACTIN-V®) reduced BV recurrence at 12 months by 15% [37].

### Vaginal *Lactobacillus* species

Bacteria belonging to the genus *Lactobacillus* are Gram-positive, aerotolerant or anaerobic bacteria named for their ability to produce lactic acid as the end product of carbohydrate metabolism [38]. Lactobacilli can exist in multiple human microbial niches, and the *Lactobacillus* species that are most common in the female genital tract, specifically *L. crispatus* and *L. iners*, will be the focus of this review. Importantly, not only does BV prevalence vary by race, but among BV-free women there are substantial race-based differences in the prevalence of these two *Lactobacillus* species. Ravel and colleagues found that the vaginal microbiota of BV-free ACB, Hispanic, and Asian women was most likely to be predominated by *L. iners* (53% of BV-free ACB women, vs. 37% *L. crispatus*, for example) [4]. In contrast, *L. crispatus* was the predominant species of *Lactobacillus* among BV-free white women (51% of BV-free white women, vs. 30% *L. iners*). Similarly, our own group found that *L. iners* predominated the vaginal microbiota in 70% of BV-free ACB women from Toronto, Canada, with just 23% demonstrating *L. crispatus* predominance [3].

Substantial biological differences exist between *Lactobacillus* species, including their genital immune impact, ideal growth conditions, production of lactic acid and specific lactate isomers, inhibition of BV-associated bacteria, adherence to epithelial cells, and resistance to antibiotics [36]. As will be discussed later, these biological differences may have important implications for HIV risk. Inter-strain biological diversity within *Lactobacillus* species also exists, but this review will focus primarily on the differential impact of common *Lactobacillus* species on HIV risk.

### Vaginal *Lactobacillus* species and HIV risk: observational findings

The clear association between BV and HIV risk has made it tempting to dichotomize the vaginal microbiota into “BV-positive” and “BV-negative” as it relates to HIV susceptibility. Early studies investigating the relationship between the vaginal microbiota and HIV risk relied on clinical criteria or a Gram’s stain to diagnose BV [39–41]. While these methods established a connection between BV and HIV risk, they were unable to draw conclusions regarding heterogeneity in HIV risk and different *Lactobacillus* species, since the latter cannot be differentiated. Therefore, based on these studies alone, one might expect all vaginal lactobacilli to provide equivalent protection against HIV acquisition. However, the advent of gene sequencing technologies means that researchers have more recently been able to evaluate the impact of specific *Lactobacillus* species on HIV risk.

Borgdorff and colleagues compared HIV/STI prevalence between women with vaginal *L. crispatus* predominance, *L. iners* predominance, and diverse microbiota states (broadly classified as molecular BV) [6]. While vaginal *L. iners* predominance was associated with a lower HIV/STI prevalence than BV in this cross-sectional study, a vaginal microbiota predominated by *L. crispatus* was associated with a lower HIV/STI prevalence than either BV or *L. iners*. Chehoud and colleagues compared the relative abundance of key vagina bacteria between HIV-positive and HIV-negative women from Chicago but did not find a significant difference in the relative abundance of either *L. iners* or *L. crispatus* [42]. However, the cross-sectional design of both studies limited the authors’ ability to assess the potential confounding effect of HIV status on the vaginal microbiota. In a prospective cohort study investigating the impact of the vaginal microbiota on HIV acquisition among initially HIV-uninfected young women from South Africa, participants with a *Lactobacillus*-deficient vaginal microbiota were at an elevated risk of HIV acquisition, but not all women with a *Lactobacillus*-predominant vaginal microbiota were equally protected [27]. Specifically, HIV risk was lower among women with *L. crispatus* predominance, while no protection was afforded by a microbiota where *L. iners* predominated [27]. In contrast, McClelland and colleagues used a longitudinal, nested case-control format to evaluate the association between the key vaginal bacteria and HIV risk among women from eastern and southern Africa [26]. Here, a higher relative abundance of *L. iners* was associated with reduced HIV risk, while the less-frequently detected L. crispatus was not; when absolute (rather than relative) bacterial abundance was assessed, neither was associated with HIV risk, and the authors hypothesized that a low relative abundance of any *Lactobacillus* species might simply serve as a marker for women with relatively higher concentrations of HIV-associated BV bacteria. Both prospective studies attempted to control their analyses for sexual behavior, which may serve as a confounding factor that alters both HIV risk and the genital microbiota, but further work is needed to prove that the genital microbiome plays a causal role in HIV susceptibility. Therefore, while the presence of BV is clearly associated with elevated HIV risk [12], from these epidemiological studies it remains unclear the degree to which different *Lactobacillus* species may play a causal role in altering HIV susceptibility.

### The mucosal immunology of HIV susceptibility

Most HIV acquisition in women occurs during or after condomless penile-vaginal sex with an HIV-infected male partner [2]. Most sexual HIV exposures are effectively repelled by multiphasic genital mucosa immune defenses that include viral binding by mucus [43], destruction by innate antimicrobial peptides [44], an intact epithelial barrier [45], and (compared to the rectal mucosa) a relative paucity of HIV-susceptible intra-epithelial and submucosal target cells [46]. However, in the context of a productive exposure, HIV can penetrate the epithelial barrier as soon as 4 h following exposure [47], with subsequent infection of several cell subsets including T cells, immature dendritic cells, Langerhans cells, and macrophages; the predominant HIV target cell during the earliest stages of mucosal infection appears to be activated CD4+ T cells expressing the co-receptor CCR5, particularly mucosal Th17 cells [46, 48–50].

Despite relatively low rates (well under 1%) of per-contact HIV acquisition after a sexual exposure in the female genital tract, numerous factors can facilitate an HIV-permissive environment in the female genital tract, including the presence of STIs, BV, and vaginal washing [12], all of which appear to increase risk through the common central pathway of inducing inflammation [5, 51, 52]. Inflammation elevates HIV risk by several mechanisms. First, genital mucosal inflammation has been linked to the suppression of genital mucus and innate antimicrobial responses in the female genital tract (FGT), including decreased innate immune factors such as secretory leukocyte protease inhibitor (SLPI) and defensins [53]. Second, elevated proinflammatory cytokines can directly disrupt the genital epithelial barrier which can facilitate passage by HIV [45, 54, 55]. Potential mechanisms by which proinflammatory cytokines disrupt the epithelial barrier include direct disruption of tight junction proteins between epithelial cells [45] and promotion of tissue remodeling at the expense of barrier function [54]. Third, inflammation induces recruitment of activated CD4+ T cell HIV targets to the genital mucosa [54]. Recruitment of HIV target cells may be mediated by the induction of proinflammatory chemokines such as IL-8, a subset of cytokines that induce chemotaxis of target cells [54]. HIV acquisition is linked to an increased pre-exposure concentration of inflammatory antimicrobial peptides such as α-defensins and cathelicidins [56], and Masson and colleagues further investigated the relationship between genital inflammation and HIV risk by measuring levels of vaginal proinflammatory cytokines and chemokines among South African women: here, HIV risk was significantly higher among women with high preceding combinatorial score of the same proinflammatory cytokines linked to epithelial barrier disruption and chemokines linked to HIV target cell recruitment [57]. In particular, women acquiring HIV had significantly higher pre-acquisition genital concentrations of the chemokines IP-10 and MIP-1b, both of which are chemoattractant for CD4+ T cells, and a trend towards higher IL-1α, the prototypical proinflammatory cytokine that has been linked to epithelial barrier disruption [57]. Other studies have found associations between elevated levels of genital proinflammatory cytokines/chemokines and HIV target cell counts [54, 58] and protein signatures of epithelial barrier disruption [54], emphasizing that genital inflammation likely enhances HIV susceptibility both via epithelial disruption and mucosal immune cell recruitment and activation.

### Vaginal microbiota and HIV risk: mechanistic considerations

#### Impact of BV-associated bacteria on genital immunology

In keeping with the impact of the genital immune milieu on HIV susceptibility, the consistent and strong associations of the vaginal microbiota with HIV risk are likely mediated in large part by microbiota-host immune interactions. BV may elevate HIV risk by inducing genital mucosal inflammation, as defined by elevated genital proinflammatory cytokines [5]. Genital epithelial and antigen-presenting cells initiate an inflammatory response to Gram-negative BV-associated bacteria, such as *Gardnerella vaginalis* and *Prevotella bivia*, upon sensing bacterial products such as lipopolysaccharide [5, 59]; Toll-like receptor signaling with subsequent activation of the NF-kB pathway in epithelial and antigen-presenting cells results in production of proinflammatory cytokines and chemokines that induce epithelial barrier disruption and lymphocyte recruitment, respectively [5, 60]. BV may also elevate HIV risk by impairing innate immune defenses to HIV, including the cervicovaginal mucus barrier, enhancing the ability of HIV to access the genital epithelium [61]. In addition, the production of metabolites such as short-chain fatty acids by BV-associated bacteria has been linked to the upregulation of proinflammatory gene pathways in vaginal epithelial cells and to the elevated production of proinflammatory cytokines [62].

#### Lactobacillus effects on genital mucosal immunology

The impact of BV-associated bacterial species on mucosal immunology has been explored extensively, but less is known about differences between *Lactobacillus* species in their mucosal immune impact. Shannon and colleagues found a direct correlation between the absolute (and relative) abundance of *Prevotella bivia* and *Gardnerella vaginalis* with genital inflammation in ACB women; in exploring *Lactobacillus* immune impact they found that the abundance of *L. crispatus* was strongly inversely associated with vaginal inflammation, while *L. iners* abundance was unrelated to genital inflammation, but strongly associated with elevated vaginal levels of the chemokine IP-10 [3]. In this cohort, potential biological confounders, including recent sex, did not differ between women with a vaginal microbiota that was predominated by non-*iners Lactobacillus* spp. vs. those with *L. iners* predominance. However, in a prospective cohort study of South African women, Anahtar and colleagues found no difference in various levels of cytokines and chemokines between women with predominance by non-*iners Lactobacillus* spp. and *L. iners* predominance after controlling for potential confounders, although levels of the chemokine IP-10 were not compared between bacterial communities [5]. Despite the lack of difference in cytokine levels between bacterial communities in vivo, subsequent in vitro analyses demonstrated elevated production of IL-8 by vaginal epithelial cells following exposure to *L. iners* and BV-associated anaerobes, but not *L. crispatus*; this proinflammatory chemokine has been linked to neutrophil recruitment and elevated HIV risk [57]. However, neither *L. crispatus* nor *L. iners* exposure elicited production of the proinflammatory cytokines IL-1α or IL-1β. Other groups have shown similar immunomodulatory effects of *L. iners* in laboratory models. Anton and colleagues demonstrated that exposure to bacteria-free supernatant from *L. iners* cultures induced IL-6 production by endocervical epithelial cells and increased endocervical and ectocervical cell permeability, while *L. crispatus* bacteria-free supernatant dampened IL-6 and IL-8 production by endocervical cells and reduced the ectocervical cell permeability induced by inflammatory stimuli [63]. In addition, Doerflinger and colleagues showed that *Atopobium vaginae* induced both the expression of proinflammatory genes by vaginal epithelial cells and the release of downstream proinflammatory cytokines (IL-6, IL-8, and TNF), in contrast to *L. crispatus* which had no effects on cytokine production or proinflammatory gene expression. Interestingly, while *L. iners* did not induce proinflammatory cytokines in the latter study, it did induce the expression of genes related to proinflammatory signaling pathways and cytokines [59].

#### The genital immune impact of microbiota-focused clinical interventions

In a recent study investigating the impact of metronidazole treatment on genital immunology in Kenyan women with BV [28], treatment resulted in a *Lactobacillus*-predominant microbiota at one month in approximately half the participants. While the prototypic proinflammatory cytokines (IL-1α/β) decreased among participants who cleared BV, several chemokines previously linked to HIV susceptibility (IP-10, MIG, MIP-3α and others) were substantially increased one month after treatment. This increase was only apparent among participants who had cleared BV, strongly suggesting that the changes had been caused by the microbial shift from BV to *Lactobacillus* predominance; however, it was unclear whether effects were due to a decrease in BV-associated bacteria, to an increase in lactobacilli, or both. Furthermore, since this study was performed in ACB women from Kenya, the great majority of participants who cleared BV then demonstrated vaginal *L. iners* predominance at follow-up, limiting the ability to define the genital immune impact of a post-BV treatment microbiota dominated by *L. crispatus*. Sultan and colleagues recently demonstrated similar results in their study of standard BV treatment and genital immunology among American and Kenyan women [64]. At approximately 1-month post-treatment, clearance of BV (defined by change in Shannon diversity index) was associated with a decrease in the proinflammatory cytokines IL-1α and IL-1β and an increase in the proinflammatory chemokines IP-10, MIG, and MCP-1. In contrast to the previous study, nearly half of the American women who were BV-negative at 1-month post-treatment had a vaginal microbiota predominated by non-*iners* lactobacilli. However, the impact of BV treatment on genital immunology based on the species of *Lactobacillus* that predominated post-treatment was not explored in more detail.

#### Vaginal lactobacilli and microbiota dynamics

Independent of any direct bacterial immune effects, lactobacilli might also confer different levels of HIV protection through their ability to exclude other microbiota components and hence to resist shifts towards a BV-type (i.e., HIV-susceptible) microbiota. While several different *Lactobacillus spp.* commonly predominate the vaginal microbiota, *L. crispatus* confers protection against subsequent BV acquisition, while *L. iners* does not [18, 65]. Longitudinal vaginal swab collection with 16S rRNA gene sequencing confirmed that women with an *L. crispatus* or *L. gasseri* predominant vaginal microbiota at baseline exhibited far fewer transitions to molecular BV than those with *L. iners* predominance [17]. Similar findings were obtained when quantitative frequencies, rather than proportional frequencies provided by 16S rRNA sequencing, were used to characterize the vaginal microbiota. In a cohort of pregnant women, *L. crispatus* and *G. vaginalis* nearly always predominated over one another when both species were present in the microbiota. *L. iners*, in contrast, rarely predominated over *G. vaginalis* and often co-occurred with the BV-associated species at similar frequencies [66]. As will be discussed later, the co-occurrence of *L. iners* with BV-associated bacteria may have important immunological implications [67]. Therefore, *L. crispatus* may confer additional protection against HIV relative to *L. iners* due to the enhanced ability of the former species to exclude BV-associated bacteria.

### *Lactobacillus* spp. and HIV risk: biochemical considerations

Distinct biochemical characteristics of *L. crispatus* and *L. iners* likely contribute to their disparate genital immune and bacterial exclusion effects. For example, all lactobacilli produce lactic acid, which contributes to immune quiescence, but the extent to which lactic acid dampens immune activation differs between *Lactobacillus* species. In vitro experiments investigating effects on cervical epithelial cells found that lactic acid dampened the production of proinflammatory cytokines induced by various stimuli but that these anti-inflammatory effects were mediated by the protonated form of lactic acid. Protonation involves the addition of protons to a molecule and is enhanced in proton-rich, low pH environments [68]. Since *L. iners* often co-exists with BV-associated bacteria while *L. crispatus* does not [66, 69], a higher pH in the context of *L. iners* may reduce protonated lactic acid and associated immunomodulatory effects. Importantly, *ex vivo* anti-HIV activity of vaginal secretions is also mediated by the protonated form of lactic acid [70].

While lactic acid can dampen inflammation in vitro, not all lactic acid stereoisomers behave in the same way or are produced equally by all *Lactobacillus* species. Lactic acid can exist as D- or L-stereoisomers, which are distinguished by the spatial arrangement of their atoms [71], and while most vaginal *Lactobacillus* species are able to produce both stereoisomers, *L. iners* can only produce the L-stereoisomer [72]. Nunn and colleagues demonstrated that it was specifically the presence of high levels of D-lactic acid that predicted HIV trapping in cervicovaginal mucus (CVM), as opposed to vaginal pH or total lactic acid concentration, and that CVM samples with high D-lactic acid were typically from women with a vaginal microbiota predominated by *L. crispatus* [73]. Overall, the promotion of lower pH through exclusion of BV-associated bacteria, as well as the production of both D- and L-lactic acid isomers, may explain the enhanced mucosal immunoregulatory effects of *L. crispatus*.

### Genetic characteristics of *Lactobacillus* spp.

Although there are substantial genetic differences between all vaginal *Lactobacillus* species, *L. iners* is unique in several ways. While *L. crispatus* and other vaginal lactobacilli have a genome of approximately 3–4 Mbp, the genome of *L. iners* is only about 1 Mbp, suggesting that *L. iners* will be more reliant on external sources for metabolic requirements [74]. A small genome size is also seen in several human symbionts and parasites, suggesting a possible parasitic role for *L. iners* compared to other vaginal *Lactobacillus* species [74]. The small genome of *L. iners* also means that it lacks the coding sequences for key metabolites such as D-lactic acid dehydrogenase [72], which is why this is the only vaginal *Lactobacillus* species unable to produce the D-isomer of lactic acid (see above). However, the small genome of *L. iners* does not mean that this species does not possess several unique genes not found in other *Lactobacillus* spp., including the toxin inerolysin, a type of cytolysin that lyses epithelial cells and erythrocytes and activates proinflammatory signaling [74, 75]. Co-occurrence of *L. iners* with BV-associated bacteria has been consistently demonstrated and may have important implications for genital immunology. Macklaim and colleagues demonstrated that *L. iners* gene expression differed substantially during bacterial growth in BV conditions, with an upregulation in the expression of cytolysin, which may contribute to epithelial disruption and inflammation [67]. Taken together, the presence and absence of specific genes that encode for immunomodulatory compounds in the genome of *L. iners* may explain why *L. iners* is less immune quiescent than other vaginal *Lactobacillus* species and may even be proinflammatory.

The unique genome of *L. iners* may be optimized for survival alongside BV-associated bacteria, coding for several stress-resistance proteins, such as the σ-factor RpoE, which promotes cell envelope integrity during stress conditions. The presence of these stress-resistance proteins suggests that *L. iners* may be optimized for survival in a fluctuating environment and therefore does not have the same requirement to resist microbial shifts as other vaginal *Lactobacillus* species [17, 74]. The σ-factor RpoE is also encoded for in the genome of Gram-negative bacteria, including several BV-associated bacteria species. Interestingly, the genome of the BV-associated bacteria species *G. vaginalis* also encodes for vaginolysin, a toxin that acts very similarly to the *L. iners*-derived toxin inerolysin [74]. Shared genetic characteristics between *L. iners* and some BV-associated bacteria indicates that they may have co-evolved to exist in a similar microbial niche.

## Conclusion

Although BV-associated bacteria have been consistently linked to elevated HIV susceptibility, vaginal *Lactobacillus* predominance and the absence of BV does not necessarily imply HIV protection. Indeed, there is now considerable evidence that *L. iners*, the predominant vaginal species globally, is at best immunologically inert and may be proinflammatory. *L. iners* does not have the same exclusionary effects on BV-associated bacteria as other vaginal *Lactobacillus* species and is unable to produce the more anti-inflammatory D-isomer of lactic acid, while *L. crispatus* confers anti-inflammatory effects in the FGT and is better able to resist microbial shifts to a BV-type state. Thus, it may be preferable to promote an *L. crispatus*-predominant vaginal microbiota, rather than the simple absence of BV, to maximize subsequent protection against HIV. Fortunately, there appears to be interest in developing novel BV treatment strategies that promote vaginal predominance of *L. crispatus* and/or other non-*iners* species of *Lactobacillus*. Evidence of this includes a recent study demonstrating that an *L. crispatus*-based live biotherapeutic (LACTIN-V®) can induce *L. crispatus* colonization and reduce BV recurrence. Future clinical trials are needed to evaluate the ability of probiotics and live biotherapeutics containing non-*iners Lactobacillus* spp. to reduce genital inflammation, exclude BV-associated bacteria, and ultimately reduce HIV incidence.

## Data Availability

Not applicable

## Abbreviations

ACB: African, Caribbean, and Black
BV: Bacterial vaginosis
CCR: Chemokine receptor
CD: Cluster of differentiation
CVM: Cervicovaginal mucus
FGT: Female genital tract
HIV: Human immunodeficiency virus
HSV-2: Herpes simplex virus type 2
IL: Interleukin
IP-10: Interferon gamma-induced protein 10
SSA: Sub-Saharan Africa
Mbp: Mega base pair
MIG: Monokine induced by gamma interferon
MIP: Macrophage inflammatory protein
MSM: Men who have sex with men
NF-kB: Nuclear factor kappa-light-chain-enhancer of activated B cells
RpoE: RNA polymerase, extracytoplasmic E
rRNA: Ribosomal ribonucleic acid
SLPI: Secretory leukocyte protease inhibitor
STI: Sexually transmitted infection
Th: T helper
TNF: Tumor necrosis factor
